# *N*-[(4-Amino-5-sulfanyl­idene-4,5-dihydro-1*H*-1,2,4-triazol-3-yl)meth­yl]-4-methyl­benzamide

**DOI:** 10.1107/S1600536810035014

**Published:** 2010-09-04

**Authors:** Hoong-Kun Fun, Chin Sing Yeap, Yatin Mange, Arun M. Isloor, Chitrakar Hegde

**Affiliations:** a X-ray Crystallography Unit, School of Physics, Universiti Sains Malaysia, 11800 USM, Penang, Malaysia; b Organic Chemistry Division, Department of Chemistry, National Institute of Technology–Karnataka, Surathkal, Mangalore 575 025, India; c Department of Chemistry, NITTE Meenakshi Institute of Technology, Yelahanka, Bangalore, India

## Abstract

In the title compound, C_11_H_13_N_5_OS, the dihedral angle between the triazole ring and the benzene ring is 84.21 (7)°. The amino group adopts a pyramidal configuration. An intra­molecular N—H⋯O hydrogen bond stabilizes the mol­ecular structure and generates an *S*(8) ring. In the crystal, mol­ecules are linked by inter­molecular N—H⋯O, N—H⋯S, N—H⋯N and C—H⋯S hydrogen bonds into layers lying parallel to the *bc* plane. The crystal structure is further stabilized by aromatic π–π stacking inter­actions [centroid–centroid distance = 3.3330 (7) Å].

## Related literature

For applications of 1,2,4-triazole derivatives, see: Demirbas *et al.* (2002 ▶, 2004 ▶); Tozkoparan *et al.* (2000 ▶); Turan-Zitouni *et al.* (1999 ▶); Kritsanida *et al.* (2002 ▶); Holla *et al.* (2002 ▶); Foroumadi *et al.* (2003 ▶); Isloor *et al.* (2009 ▶); Shujuan *et al.* (2004 ▶); Verreck *et al.* (2003 ▶); Clemons *et al.* (2004 ▶). For related structures, see: Fun *et al.* (2009**a* ▶,b* ▶). For stability of the temperature controller used in the data collection, see: Cosier & Glazer (1986 ▶).
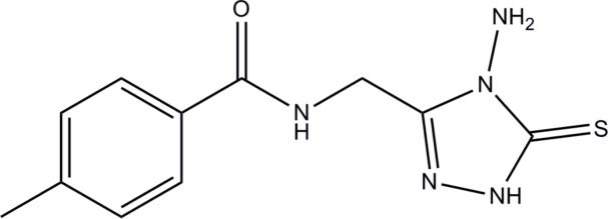


## Experimental

### 

#### Crystal data

C_11_H_13_N_5_OS
*M* *_r_* = 263.32
Monoclinic, 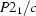

*a* = 14.8148 (1) Å
*b* = 8.6702 (1) Å
*c* = 9.8534 (1) Å
β = 104.923 (1)°
*V* = 1222.96 (2) Å^3^
*Z* = 4
Mo *K*α radiation
μ = 0.26 mm^−1^
*T* = 100 K
0.30 × 0.27 × 0.11 mm

#### Data collection

Bruker SMART APEXII CCD diffractometer
Absorption correction: multi-scan (*SADABS*; Bruker, 2009 ▶) *T*_min_ = 0.926, *T*_max_ = 0.972
15018 measured reflections
3555 independent reflections
3142 reflections with *I* > 2σ(*I*)
*R*_int_ = 0.023

#### Refinement

*R*[*F* ^2^ > 2σ(*F* ^2^)] = 0.035
*wR*(*F* ^2^) = 0.093
*S* = 1.07
3555 reflections
180 parameters
H atoms treated by a mixture of independent and constrained refinement
Δρ_max_ = 0.44 e Å^−3^
Δρ_min_ = −0.28 e Å^−3^

### 

Data collection: *APEX2* (Bruker, 2009 ▶); cell refinement: *SAINT* (Bruker, 2009 ▶); data reduction: *SAINT*; program(s) used to solve structure: *SHELXTL* (Sheldrick, 2008 ▶); program(s) used to refine structure: *SHELXTL*; molecular graphics: *SHELXTL*; software used to prepare material for publication: *SHELXTL* and *PLATON* (Spek, 2009 ▶).

## Supplementary Material

Crystal structure: contains datablocks global, I. DOI: 10.1107/S1600536810035014/hb5629sup1.cif

Structure factors: contains datablocks I. DOI: 10.1107/S1600536810035014/hb5629Isup2.hkl

Additional supplementary materials:  crystallographic information; 3D view; checkCIF report

## Figures and Tables

**Table 1:** Hydrogen-bond geometry (Å, °)

*D*—H⋯*A*	*D*—H	H⋯*A*	*D*⋯*A*	*D*—H⋯*A*
N1—H1*N*1⋯O1^i^	0.841 (18)	2.187 (17)	2.9824 (14)	157.7 (17)
N3—H1*N*3⋯S1^ii^	0.851 (19)	2.524 (19)	3.2168 (11)	139.2 (15)
N3—H1*N*3⋯N5^ii^	0.851 (19)	2.276 (18)	2.9738 (15)	139.3 (16)
N5—H1*N*5⋯O1	0.843 (19)	2.169 (18)	2.9587 (15)	155.9 (16)
N5—H2*N*5⋯S1^iii^	0.880 (18)	2.666 (18)	3.5381 (12)	171.2 (14)
C8—H8*A*⋯S1^iv^	0.97	2.87	3.4456 (12)	119

Symmetry codes: (i) 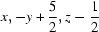; (ii) 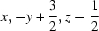; (iii) 
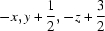
; (iv) 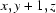.

**Fig. 1.**
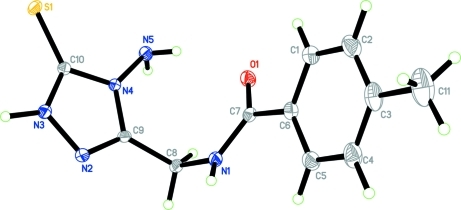
The molecular structure of the title compound with 50% probability ellipsoids for non-H atoms.

**Fig. 2.**
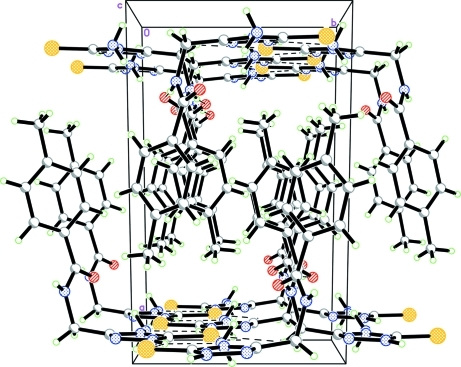
The crystal packing of (I), viewed down the c axis, showing molecular planes parallel to bc plane. Intermolecular hydrogen bonds are shown as dashed lines.

## supplementary crystallographic information

### Comment

The synthesis of 1,2,4-triazole derivatives has been attracting widespread attention due to their diverse biological activities such as antimicrobial, anti-inflammatory, and analgesic antitumorial activities (Demirbas *et al*., 2004; Tozkoparan *et al.*, 2000; Turan-Zitouni *et al.*, 1999; Demirbas *et al.*, 2002; Kritsanida *et al.*, 2002; Holla *et al.*, 2002; Foroumadi *et al.*, 2003; Isloor *et al.*, 2009). There are some antimicrobial drugs containing a triazole moiety. For instance, fluconazole and itraconazole are used in medical therapy (Shujuan *et al.*, 2004; Verreck *et al.*, 2003). In addition, Vorozole, Letrozole, Fadrozole, and Anastrozole are nonsteroidal drugs used for the treatment of estrogen-dependent breast cancer (Clemons *et al.*, 2004). Prompted by the diverse activities of 1,2,4-triazole derivatives, we have synthesized the title compound to study its crystal structure.

The geometric parameters of the title compound (Fig. 1) are comparable to its related structures (Fun *et al.*, 2009**a*, b*).The dihedral angle between the triazole ring [C9/N2/N3/C10/N4] and the benzene ring [C1–C6] is 84.21 (7)°. The amino group adopts a pyramidal configuration. An intramolecular N5—H1N5···O1 hydrogen bond stabilizes the molecular structure. In the crystal structure, the molecules are linked by intermolecular N1—H1N1···O1, N3—H1N3···S1, N3—H1N3···N5, N5—H2N5···S1 and C8—H8A···S1 hydrogen bonds (Table 1) into two-dimensional planes parallel to *bc* plane (Fig. 2). *Cg*1···*Cg*1 of 3.3330 (7) Å (-*x*, 2 - *y*, 1 - *z*) interactions further stabilize the crystal structure. *Cg*1 is centroid of the triazole ring.

### Experimental

A mixture of {[(4-methylphenyl)carbonyl]amino}acetic acid (1.93 g, 0.01 mol) and thiocarbohydrazide (1.0 g, 0.01 mol) was taken in a round bottomed flask fitted with a reflux condenser. The mixture was then fused on oil bath for 1 h. The reaction mass was slowly cooled to room temperature and the solid mass was treated with NaHCO_3_ solution. The separated solid was collected by filtration and dried. Recrystallization was done from a mixture of ethanol-dioxane to yield colourless blocks of (I). Yield: 2.0 g, 76.0%, *m.p.* = 493–494 K.

### Refinement

N-bound hydrogen atoms were located from the difference Fourier map and refined freely. The rest of the hydrogen atoms were positioned geometrically and refined using a riding model. A rotating group model was used for the methyl group.

### Crystal data

**Table:**

C_11_H_13_N_5_OS	*F*(000) = 552
*M**_r_* = 263.32	*D*_x_ = 1.430 Mg m^−^^3^
Monoclinic, *P*2_1_/*c*	Mo *K*α radiation, λ = 0.71073 Å
Hall symbol: -P 2ybc	Cell parameters from 6908 reflections
*a* = 14.8148 (1) Å	θ = 2.8–32.6°
*b* = 8.6702 (1) Å	µ = 0.26 mm^−^^1^
*c* = 9.8534 (1) Å	*T* = 100 K
β = 104.923 (1)°	Block, colourless
*V* = 1222.96 (2) Å^3^	0.30 × 0.27 × 0.11 mm
*Z* = 4	

### Data collection

**Table:**

Bruker SMART APEXII CCD diffractometer	3555 independent reflections
Radiation source: fine-focus sealed tube	3142 reflections with *I* > 2σ(*I*)
graphite	*R*_int_ = 0.023
φ and ω scans	θ_max_ = 30.0°, θ_min_ = 2.8°
Absorption correction: multi-scan (*SADABS*; Bruker, 2009)	*h* = −20→20
*T*_min_ = 0.926, *T*_max_ = 0.972	*k* = −12→11
15018 measured reflections	*l* = −13→13

### Refinement

**Table:**

Refinement on *F*^2^	Primary atom site location: structure-invariant direct methods
Least-squares matrix: full	Secondary atom site location: difference Fourier map
*R*[*F*^2^ > 2σ(*F*^2^)] = 0.035	Hydrogen site location: inferred from neighbouring sites
*wR*(*F*^2^) = 0.093	H atoms treated by a mixture of independent and constrained refinement
*S* = 1.07	*w* = 1/[σ^2^(*F*_o_^2^) + (0.0404*P*)^2^ + 0.7537*P*] where *P* = (*F*_o_^2^ + 2*F*_c_^2^)/3
3555 reflections	(Δ/σ)_max_ = 0.001
180 parameters	Δρ_max_ = 0.44 e Å^−^^3^
0 restraints	Δρ_min_ = −0.28 e Å^−^^3^

### Special details

**Table:**

Experimental. The crystal was placed in the cold stream of an Oxford Cryosystems Cobra open-flow nitrogen cryostat (Cosier & Glazer, 1986) operating at 100.0 (1) K.
Geometry. All e.s.d.'s (except the e.s.d. in the dihedral angle between two l.s. planes) are estimated using the full covariance matrix. The cell e.s.d.'s are taken into account individually in the estimation of e.s.d.'s in distances, angles and torsion angles; correlations between e.s.d.'s in cell parameters are only used when they are defined by crystal symmetry. An approximate (isotropic) treatment of cell e.s.d.'s is used for estimating e.s.d.'s involving l.s. planes.
Refinement. Refinement of *F*^2^ against ALL reflections. The weighted *R*-factor *wR* and goodness of fit *S* are based on *F*^2^, conventional *R*-factors *R* are based on *F*, with *F* set to zero for negative *F*^2^. The threshold expression of *F*^2^ > σ(*F*^2^) is used only for calculating *R*-factors(gt) *etc*. and is not relevant to the choice of reflections for refinement. *R*-factors based on *F*^2^ are statistically about twice as large as those based on *F*, and *R*- factors based on ALL data will be even larger.

### Fractional atomic coordinates and isotropic or equivalent isotropic displacement parameters (Å^2^)

**Table:**

	*x*	*y*	*z*	*U*_iso_*/*U*_eq_	
S1	0.10394 (2)	0.62450 (3)	0.66661 (3)	0.01567 (9)	
O1	0.25236 (6)	1.16404 (11)	0.93553 (9)	0.01636 (19)	
N1	0.22250 (7)	1.27102 (12)	0.71802 (11)	0.0132 (2)	
N2	0.10834 (7)	1.02977 (12)	0.49330 (11)	0.0126 (2)	
N3	0.10360 (7)	0.87126 (12)	0.49292 (11)	0.0123 (2)	
N4	0.10800 (7)	0.93784 (11)	0.70210 (10)	0.01071 (19)	
N5	0.10633 (8)	0.92596 (13)	0.84341 (11)	0.0144 (2)	
C1	0.44707 (9)	1.19963 (16)	0.96321 (14)	0.0194 (3)	
H1A	0.4293	1.1194	1.0134	0.023*	
C2	0.54098 (10)	1.23546 (18)	0.98378 (16)	0.0242 (3)	
H2A	0.5857	1.1781	1.0474	0.029*	
C3	0.56933 (10)	1.35571 (18)	0.91096 (15)	0.0244 (3)	
C4	0.50110 (10)	1.44261 (18)	0.81922 (15)	0.0244 (3)	
H4A	0.5188	1.5258	0.7724	0.029*	
C5	0.40675 (9)	1.40711 (16)	0.79637 (14)	0.0196 (3)	
H5A	0.3620	1.4655	0.7338	0.024*	
C6	0.37934 (9)	1.28382 (14)	0.86742 (13)	0.0147 (2)	
C7	0.27951 (8)	1.23533 (14)	0.84421 (13)	0.0130 (2)	
C8	0.12391 (8)	1.22850 (13)	0.67896 (13)	0.0126 (2)	
H8A	0.0896	1.2996	0.6084	0.015*	
H8B	0.0990	1.2362	0.7606	0.015*	
C9	0.11102 (8)	1.06742 (13)	0.62240 (12)	0.0110 (2)	
C10	0.10403 (8)	0.81094 (13)	0.61801 (12)	0.0115 (2)	
C11	0.67179 (11)	1.3904 (2)	0.9308 (2)	0.0371 (4)	
H11A	0.7038	1.3810	1.0284	0.056*	
H11B	0.6789	1.4936	0.8996	0.056*	
H11C	0.6978	1.3188	0.8769	0.056*	
H1N1	0.2453 (12)	1.299 (2)	0.6521 (18)	0.020 (4)*	
H1N3	0.1054 (13)	0.822 (2)	0.419 (2)	0.031 (5)*	
H1N5	0.1532 (13)	0.975 (2)	0.8901 (19)	0.025 (5)*	
H2N5	0.0541 (12)	0.970 (2)	0.8501 (17)	0.016 (4)*	

### Atomic displacement parameters (Å^2^)

**Table:**

	*U*^11^	*U*^22^	*U*^33^	*U*^12^	*U*^13^	*U*^23^
S1	0.02516 (17)	0.00896 (14)	0.01334 (15)	−0.00100 (11)	0.00578 (12)	−0.00009 (10)
O1	0.0186 (4)	0.0182 (4)	0.0122 (4)	−0.0036 (3)	0.0038 (3)	0.0004 (3)
N1	0.0147 (5)	0.0127 (5)	0.0120 (5)	−0.0028 (4)	0.0030 (4)	0.0000 (4)
N2	0.0135 (5)	0.0108 (4)	0.0127 (5)	−0.0002 (4)	0.0020 (4)	0.0006 (4)
N3	0.0154 (5)	0.0106 (4)	0.0112 (5)	0.0000 (4)	0.0039 (4)	−0.0009 (4)
N4	0.0127 (4)	0.0094 (4)	0.0094 (4)	−0.0003 (3)	0.0018 (3)	0.0000 (3)
N5	0.0197 (5)	0.0143 (5)	0.0098 (5)	−0.0010 (4)	0.0046 (4)	−0.0009 (4)
C1	0.0188 (6)	0.0188 (6)	0.0193 (6)	−0.0019 (5)	0.0023 (5)	−0.0002 (5)
C2	0.0160 (6)	0.0269 (7)	0.0259 (7)	0.0013 (5)	−0.0014 (5)	−0.0030 (6)
C3	0.0165 (6)	0.0337 (8)	0.0237 (7)	−0.0067 (5)	0.0063 (5)	−0.0097 (6)
C4	0.0233 (7)	0.0312 (7)	0.0192 (7)	−0.0125 (6)	0.0063 (5)	−0.0019 (5)
C5	0.0203 (6)	0.0221 (6)	0.0155 (6)	−0.0047 (5)	0.0026 (5)	0.0016 (5)
C6	0.0150 (5)	0.0155 (5)	0.0138 (5)	−0.0019 (4)	0.0038 (4)	−0.0025 (4)
C7	0.0153 (5)	0.0105 (5)	0.0132 (5)	−0.0002 (4)	0.0034 (4)	−0.0018 (4)
C8	0.0130 (5)	0.0100 (5)	0.0143 (5)	0.0006 (4)	0.0024 (4)	−0.0006 (4)
C9	0.0106 (5)	0.0097 (5)	0.0121 (5)	0.0004 (4)	0.0016 (4)	0.0015 (4)
C10	0.0114 (5)	0.0112 (5)	0.0114 (5)	0.0007 (4)	0.0019 (4)	−0.0001 (4)
C11	0.0182 (7)	0.0538 (11)	0.0405 (10)	−0.0117 (7)	0.0099 (7)	−0.0131 (8)

### Geometric parameters (Å, °)

**Table:**

S1—C10	1.6860 (12)	C1—H1A	0.9300
O1—C7	1.2409 (15)	C2—C3	1.390 (2)
N1—C7	1.3469 (16)	C2—H2A	0.9300
N1—C8	1.4587 (15)	C3—C4	1.391 (2)
N1—H1N1	0.843 (18)	C3—C11	1.510 (2)
N2—C9	1.3040 (15)	C4—C5	1.3919 (19)
N2—N3	1.3761 (14)	C4—H4A	0.9300
N3—C10	1.3375 (15)	C5—C6	1.3942 (18)
N3—H1N3	0.85 (2)	C5—H5A	0.9300
N4—C10	1.3695 (15)	C6—C7	1.4974 (17)
N4—C9	1.3780 (14)	C8—C9	1.4975 (16)
N4—N5	1.4028 (14)	C8—H8A	0.9700
N5—H1N5	0.84 (2)	C8—H8B	0.9700
N5—H2N5	0.880 (17)	C11—H11A	0.9600
C1—C2	1.3885 (19)	C11—H11B	0.9600
C1—C6	1.3938 (18)	C11—H11C	0.9600
			
C7—N1—C8	122.19 (10)	C4—C5—H5A	120.0
C7—N1—H1N1	119.9 (12)	C6—C5—H5A	120.0
C8—N1—H1N1	116.3 (12)	C1—C6—C5	119.31 (12)
C9—N2—N3	103.97 (9)	C1—C6—C7	117.83 (11)
C10—N3—N2	113.57 (10)	C5—C6—C7	122.85 (12)
C10—N3—H1N3	126.8 (14)	O1—C7—N1	122.72 (11)
N2—N3—H1N3	119.4 (14)	O1—C7—C6	121.40 (11)
C10—N4—C9	108.24 (10)	N1—C7—C6	115.87 (10)
C10—N4—N5	122.18 (10)	N1—C8—C9	110.91 (9)
C9—N4—N5	129.57 (10)	N1—C8—H8A	109.5
N4—N5—H1N5	106.3 (12)	C9—C8—H8A	109.5
N4—N5—H2N5	106.4 (11)	N1—C8—H8B	109.5
H1N5—N5—H2N5	110.9 (17)	C9—C8—H8B	109.5
C2—C1—C6	120.09 (13)	H8A—C8—H8B	108.0
C2—C1—H1A	120.0	N2—C9—N4	110.76 (10)
C6—C1—H1A	120.0	N2—C9—C8	124.43 (10)
C1—C2—C3	121.12 (14)	N4—C9—C8	124.61 (10)
C1—C2—H2A	119.4	N3—C10—N4	103.45 (10)
C3—C2—H2A	119.4	N3—C10—S1	129.53 (9)
C2—C3—C4	118.42 (13)	N4—C10—S1	126.98 (9)
C2—C3—C11	120.65 (15)	C3—C11—H11A	109.5
C4—C3—C11	120.93 (15)	C3—C11—H11B	109.5
C3—C4—C5	121.10 (13)	H11A—C11—H11B	109.5
C3—C4—H4A	119.4	C3—C11—H11C	109.5
C5—C4—H4A	119.4	H11A—C11—H11C	109.5
C4—C5—C6	119.90 (13)	H11B—C11—H11C	109.5
			
C9—N2—N3—C10	−0.49 (13)	C5—C6—C7—N1	25.57 (17)
C6—C1—C2—C3	0.5 (2)	C7—N1—C8—C9	−84.82 (13)
C1—C2—C3—C4	1.7 (2)	N3—N2—C9—N4	−0.06 (12)
C1—C2—C3—C11	−178.05 (14)	N3—N2—C9—C8	175.06 (10)
C2—C3—C4—C5	−2.4 (2)	C10—N4—C9—N2	0.57 (13)
C11—C3—C4—C5	177.38 (14)	N5—N4—C9—N2	−177.73 (11)
C3—C4—C5—C6	0.8 (2)	C10—N4—C9—C8	−174.55 (11)
C2—C1—C6—C5	−2.1 (2)	N5—N4—C9—C8	7.15 (19)
C2—C1—C6—C7	177.06 (12)	N1—C8—C9—N2	−82.22 (14)
C4—C5—C6—C1	1.5 (2)	N1—C8—C9—N4	92.24 (13)
C4—C5—C6—C7	−177.69 (12)	N2—N3—C10—N4	0.81 (13)
C8—N1—C7—O1	0.08 (18)	N2—N3—C10—S1	−177.08 (9)
C8—N1—C7—C6	178.70 (10)	C9—N4—C10—N3	−0.81 (12)
C1—C6—C7—O1	25.05 (17)	N5—N4—C10—N3	177.64 (10)
C5—C6—C7—O1	−155.80 (13)	C9—N4—C10—S1	177.16 (9)
C1—C6—C7—N1	−153.59 (12)	N5—N4—C10—S1	−4.39 (17)

### Hydrogen-bond geometry (Å, °)

**Table:**

*D*—H···*A*	*D*—H	H···*A*	*D*···*A*	*D*—H···*A*
N1—H1N1···O1^i^	0.841 (18)	2.187 (17)	2.9824 (14)	157.7 (17)
N3—H1N3···S1^ii^	0.851 (19)	2.524 (19)	3.2168 (11)	139.2 (15)
N3—H1N3···N5^ii^	0.851 (19)	2.276 (18)	2.9738 (15)	139.3 (16)
N5—H1N5···O1	0.843 (19)	2.169 (18)	2.9587 (15)	155.9 (16)
N5—H2N5···S1^iii^	0.880 (18)	2.666 (18)	3.5381 (12)	171.2 (14)
C8—H8A···S1^iv^	0.97	2.87	3.4456 (12)	119

Symmetry codes: (i) *x*, −*y*+5/2, *z*−1/2; (ii) *x*, −*y*+3/2, *z*−1/2; (iii) −*x*, *y*+1/2, −*z*+3/2; (iv) *x*, *y*+1, *z*.
